# Remote Monitoring of Algorithm-Detected Compensation Cues During Long-Term Home-Based Upper-Limb Training After Stroke: A Case Series

**DOI:** 10.3390/s26206651

**Published:** 2026-10-21

**Authors:** Dmitry V. Skvortsov, Danila A. Lobunko, Vera R. Petrosyan, Vladislav A. Agapov, Artem E. Pavlikov, Mikhail G. Gorodnichev

**Affiliations:** 1 Federal Center of Brain Research and Neurotechnologies of the Federal Medical Biological Agency (FMBA of Russia), Ostrovityanova St. 1, Bldg. 10, Moscow 117513, Russia; doctorlobunko@gmail.com; 2 Research and Clinical Centre of Moscow, Moscow 107031, Russia; 3 Faculty of Information Technologies, Moscow Technical University of Communications and Informatics (MTUCI), Aviamotornaya St. 8A, Moscow 111024, Russia; v.r.petrosyan@mtuci.ru (V.R.P.); v.a.agapov@mtuci.ru (V.A.A.); a.e.pavlikov@mtuci.ru (A.E.P.); m.g.gorodnichev@mtuci.ru (M.G.G.)

**Keywords:** telerehabilitation, stroke rehabilitation, upper limb, compensatory movement patterns, remote monitoring, home-based training, markerless motion analysis, human pose estimation, real-time feedback, case series

## Abstract

This retrospective case series examined whether a webcam application can monitor algorithm-detected compensation cues during long-term home upper-limb training after stroke. Rules on MediaPipe landmarks, not validated in patients, trigger real-time cues for elbow flexion, shoulder elevation, lateral trunk lean, and head tilt. In a technical check on 621 clinician-labelled repetitions by healthy adults, the 90° abduction rules were specific but missed most simulated compensations. Six patients with post-stroke hemiparesis, selected for long, regular records, trained at home on 33–96% of days over 3.8–9.9 months. The primary outcome, the cue rate (share of repetitions with the cue), was modelled per patient across 2277 control sessions (first daily executions of 180° and 90° abduction and a hand-to-mouth task). The elbow cue was most frequent in 180° abduction and the shoulder cue in the hand-to-mouth task. Only four of 1s6 categorised leading-cue trajectories (all elbow cue) held in every applicable sensitivity analysis; their directions differed. Long-term markerless home monitoring of the cues was feasible in these selected patients. Cue rates reflect pace, cue priority, and programme adjustment, and clinical assessment was not repeated, so their changes are not evidence of clinical improvement or deterioration; validation in patients and controlled studies should follow.

## 1. Introduction

Stroke, a leading cause of death and long-term disability, accounted for about 12 million new cases and 160 million disability-adjusted life years (DALYs) in 2021, according to the World Health Organization (WHO) and global burden-of-disease estimates [1,2]. Rehabilitation has to continue after discharge and at home [3,4]. Upper-limb dysfunction affects 50–80% of patients acutely, often persists into the chronic stage, and limits activities of daily living and self-care [5,6]. Inpatient rehabilitation, though intensive, is often insufficient in volume, hence the development of home-based programmes and telerehabilitation [4].

Telerehabilitation after stroke does not differ from in-person care for daily activity (moderate-quality evidence) and is comparable for upper-limb function (lower-quality evidence) [4,7]. In a randomised trial, home-based telerehabilitation was non-inferior to dose-equivalent in-clinic therapy for arm motor function [8]. A home-based system was tested in a single-group study of 12 patients over 28 days [9]. Self-administered programmes such as the Graded Repetitive Arm Supplementary Program (GRASP) improve arm function [10], and in a meta-analysis of randomised trials, home-based upper-limb interventions outperformed conventional therapy for upper-limb function and perceived arm use [11]. Most such solutions assess outcomes with clinical scales and monitor the volume of training, not movement quality. No tele-assessment of upper-limb motor function and activity after stroke yet meets the criteria for unrestricted clinical use [12].

Clinical scales serve stratification and outcome tracking but are episodic and poorly describe the kinematics and compensatory strategies of specific tasks [13]. Despite deficits in interjoint coordination, patients after stroke often reach the movement goal by compensating with trunk involvement, shoulder-girdle elevation, or elbow flexion that alters the trajectory. These compensations and their combinations, not only task success, need to be identified over time [14]. Marker-based, inertial and depth (RGB-D) systems need specialised equipment, set-up and staff time, limiting home use [15,16]. Markerless human pose estimation derives a skeletal model and kinematic features from video [16,17,18,19], but clinical use requires assessment criteria and validation on often small-amplitude rehabilitation movements [16,17].

In a systematic review of 72 post-stroke compensation studies, 35% used body-worn sensors (mostly inertial sensors and accelerometers), 33% marker-based motion capture and 22% markerless vision; most recognised one compensatory posture at a time, and only ten body-worn-sensor studies and one camera study took place in patients’ homes [20]. Inertial networks register trunk, shoulder and wrist involvement [21] and approach optical motion capture on quality metrics [22], but are expensive and cumbersome to calibrate. Wrist accelerometry, best suited to the home, measures mainly the volume of arm use; it correlates with the overall amount of compensatory movement but does not identify specific compensations [23,24].

Depth cameras (Microsoft Kinect, Microsoft Corporation, Redmond, WA, USA), the first markerless generation, were applied mainly to upper-limb rehabilitation [25]. In healthy adults reaching with simulated or induced compensation, Kinect measured trunk compensation validly and reliably, but shoulder and elbow angles with mixed results [26,27]. Kinect systems similar in principle to our application detected in real time forward trunk flexion and rotation during reaching in healthy adults [28], or lateral trunk lean, shoulder-girdle elevation and elbow flexion [29]. Home deployments were short and small, for example, four participants with chronic stroke over six weeks [30].

Standard RGB cameras with pose estimation are regarded as a path to objective assessment of post-stroke motor function on consumer devices [31]. In three elite boxers, a ten-camera markerless system followed the upper-limb joint centres to within about 2–3 cm of a marker-based reference, but joint angles differed by 6–23° (root-mean-square), largest in internal/external rotation [32]. In a monocular two-dimensional study, MediaPipe Hands, version 0.4.1675469240, tracked the index fingertip during a tracing task in ten healthy young adults; after rescaling and alignment, its error against a touchscreen lay within the study’s equivalence bound. Its authors suggested, without testing it, that such tracking suits within-person change better than between-person comparison [33]. This is in line with our restriction to within-patient comparisons.

MediaPipe Pose with an XGBoost classifier distinguished compensations during seated reaching [34]. With MediaPipe BlazePose and webcam recordings of a drinking task, shoulder compensation in 13 of 20 recorded patients was classified with accuracies of 0.83–0.95 within patients but 0.43–0.63 in unseen patients (hand-crafted kinematic features) and at chance level with raw keypoints [35]. Commercial platforms (SWORD Health, Kaia Health, MindMotion, Neofect) use inertial sensors or a camera, some with feedback on several compensations at once. Automated exercise assessment is still divided into discrete, threshold-based (rule-based) and template-based approaches [36]. Monitoring of specific compensatory patterns over months of home training, and of their course within each patient, has hardly been examined.

We developed a browser-based application for home upper-limb training: MediaPipe pose estimation on the patient’s computer checks for four compensatory patterns (lateral trunk lean, shoulder elevation, elbow flexion, and head tilt) by threshold rules and shows a real-time text cue; only per-step records reach the physician, who can adjust the programme remotely. A later detector built on the same approach was validated against two clinicians’ ratings in healthy adults simulating compensations [37]. The deployed rules, with thresholds set in advance on physicians’ reference videos, have not been validated in patients; here we also report a technical check of the rules for 90° abduction in healthy adults. The outcomes are therefore algorithm-detected compensation cues (hereafter cues), not confirmed compensatory movements.

In six patients with post-stroke hemiparesis, we aimed to describe long-term home use of this remote training and monitoring system and to model, within each patient, the dynamics of these cues. We report (i) training volume and adherence; (ii) which cues the system showed in three exercises, and how often; and (iii), as the primary objective, how the rate of the leading cue of each exercise (the primary outcome, the cue rate: the share of repetitions with the cue; Section 2.6) changed within each patient, with the time per repetition as the secondary outcome, and how robust these trajectories are to the analytical definitions. The efficacy of the training was not assessed.

## 2. Materials and Methods

### 2.1. Study Design and Setting

We present a case series on the remote monitoring of algorithm-detected compensation cues in six patients with post-stroke hemiparesis who trained at home with a web application that checks for compensatory movement patterns by threshold rules and gives real-time text cues (Figure 1). Measurements were repeated within each patient over 3.8–9.9 months between November 2024 and December 2025 (Section 2.2). The study was a single-centre longitudinal observational study without a control group or randomisation; patients were included after ethics approval. Its aim was to assess whether markerless pose estimation can serve the long-term remote monitoring of home upper-limb training and the individual adjustment of the programme. Here, long-term means observation windows of months; home-based training, sessions performed at home without a clinician present; remote monitoring, that only the application’s per-step records reach the physician; and compensation cues, the output of the four threshold rules (Section 2.4), not clinically rated compensatory movement. The report concerns clinical feasibility in the six selected patients, not system development, technical validation, or efficacy: no efficacy assessment was planned or made. The only technical analysis, a check of the 90° cue rules on clinician-labelled repetitions of healthy adults (Section 2.4), is not a validation and involved no patients. The analysis of the logs is retrospective; its outcomes were defined in the analysis plan of September 2026.

The institution provides no repeat examination after discharge, and none was planned or performed; the scales describe the baseline only, and there is no clinical outcome. The approved protocol specified neither the outcomes analysed here, nor a training duration, nor a control session. Reporting follows the Strengthening the Reporting of Observational Studies in Epidemiology (STROBE) statement, its extension for routinely collected data (RECORD) [38,39], and, for the programme, the Template for Intervention Description and Replication (TIDieR) [40].

The analysis plan, including the category thresholds, was fixed before the final analysis and was not pre-registered. The 90° exercise and the time per repetition were added to the plan after the preliminary results; one reason the plan records for the 90° exercise is that its elbow cue, unlike the 180° one, was not near the ceiling.

### 2.2. Participants

Six adults with post-stroke hemiparesis were analysed (Table 1). Patients were included if able to follow an individually selected home programme with remote monitoring, judged from the movements possible, understanding of instructions, general condition and exercise tolerance, without fixed thresholds or instruments. Recurrent stroke, a vertebrobasilar lesion, marked distal paresis, spasticity, aphasia and impaired deep sensation were not in themselves grounds for non-inclusion. A computer with a webcam was required; independent use of the application and resolution of technical problems were not (Section 2.3). The ethics approval of 12 April 2024 covers up to 20 outpatients aged 19–75 years with post-stroke upper-limb hemiparesis from April 2024 to September 2026, with unwillingness, inability to keep to the regimen, and technical impossibility of taking part as exclusion criteria. Eighteen patients trained at home with the application; the other 12 were not included because of technical difficulties at home, a short or irregular series, dropping out, or no control sessions in the programme’s sense (‘the first training’ of the day; Section 2.5). The treating physicians (D.V.S. and D.A.L.) selected the six purposively, not consecutively, for long, regular series; the numbers screened, eligible, declining and enrolled, and the number not included per reason, are not available. An instructed relative read the text cues aloud to P5 (sensorimotor aphasia). P1 (aphasia) and P2 (left-sided neglect on neuropsychological examination; reduced vision) could read at discharge; whether they read the cues is not known. P3, P4 and P6 had neither aphasia nor neglect. Sensation, cognition and vision were not scored; previous experience with computers, the home internet connection and, except for P5, help from a relative during training were not recorded.

Four patients were men; the mean age at discharge was 55.2 ± 8.6 years (mean ± standard deviation; range 40–65). All index strokes were ischaemic (one woman also had an earlier haemorrhagic stroke; her time since stroke and side refer to the index stroke), all six patients were right-handed, and the months of Table 1 run to discharge (median 10, range 3–16). The phase at discharge (Table 1) was classified by the Stroke Recovery and Rehabilitation Roundtable definitions [41]. On the first day of the window, the time since stroke had a median of 14.2 months (range 4.8–23.7); P2 passed 6 months about five weeks into the window, and the others were chronic throughout.

The baseline examination was performed at discharge by a specialist in physical rehabilitation with three years’ experience with the Action Research Arm Test (ARAT), who helped compose the inpatient but not the home programme. It comprised the ARAT [42]; Medical Research Council (MRC) strength [43] and Modified Ashworth tone [44,45] of shoulder abduction, elbow flexion and wrist extension; the modified Rankin Scale (mRS) [46]; a visual analogue scale for pain [47]; and the International Classification of Functioning, Disability and Health (ICF) category d440, fine hand use, with performance and capacity qualifiers [48] (ranges and directions: Table 1). The Fugl–Meyer Assessment and the Motor Activity Log were not performed, and no pain or fatigue scale was used after discharge. The baseline assessment preceded the first stored cue record of every patient (Section 2.5), so no cue count could have influenced it. The scores are taken from the baseline assessment sheet, with the exceptions marked in Table 1. No strength grade exceeded 3, the median ARAT score was 8 (range 0–32), and the mRS grade was 3 in five patients and 4 in P5. No adverse reactions were registered during home training by the support service or in the post-session survey, which only P2 and P6 answered regularly; the database has no field for them. No data are available on concomitant rehabilitation, medication, botulinum toxin, or other upper-limb treatment, or hospital admissions during the windows.

#### Observation Windows

The observation windows were fixed per patient in the analysis plan (Section 2.1) and were not adjusted; they begin 54–356 days after discharge. Five patients had used the application before the window: P1, P2, P3 and P6 from 4 to 11 days after discharge (43–142 days before the window) and P4 from 74 days before discharge (180 days before the window); P5 began on the first day of the window. In P1, P2, P3 and P6 the window begins within two days of a new batch of scheduled sessions, that is, of a programme review (Section 2.3). Earlier cue records (up to 81 control sessions per exercise) were not analysed, so for five patients the series does not begin with the first exposure to the cues.

For P1, P2, P5, and P6, the window ends on the date of a data export: a data cut-off, not a clinical event. Training continued and is analysed in sensitivity analysis 7 (SA7; Section 2.7). P4’s window ends with the last recorded session; participation could be ended at any time, and P4 declined to continue. P3’s ends with the last session containing the analysed exercises (29 September 2025, day 300). The batch of the programme containing them ended on 30 September 2025 and was not renewed; from 1 October 2025 he trained only with a programme containing their bilateral variants (separate exercises, not analysed).

Data after the window up to 31 August 2026 (within the approved period) are used only in SA7 and to describe use after the window.

### 2.3. Home Training Programme and the Application

The programme was individual, and the patients and relatives learnt to use the browser-based application in the clinic before discharge; all six used laptops with a built-in camera. A written memo asked for a mains-connected computer, lighting without backlighting, light-coloured clothing, 1.5–2 m from the camera with the head to the knees in the frame, and nobody else in the frame; a support service kept in contact to resolve technical questions, and video calls supported the patients, without a clinician watching them exercise. The number, timing, and content of these contacts were not logged, and no instrument for patient or caregiver burden or acceptability was used; the optional post-session survey (below) is the only self-report in the database. The camera position and angle, distance, lighting, clothing, and room were neither recorded nor checked, so the constancy of conditions between and within patients is unknown.

A session is a sequence of steps from a training template, each step being one exercise with a prescribed number of repetitions *R* and a time limit, or a rest; sessions had a median of 13 exercise steps (per-patient medians 9–31). Templates are graded by level; an assigned template is a course; 19 templates were used in the windows (five to ten per patient, of which two to four contributed control sessions). Sessions are scheduled in advance as assignments, created in batches (Section 2.6). On 43–100% of a patient’s active days, two or more templates were used, so the template of the control session depends on which session came first.

The rehabilitation physician was to review the programme monthly, based on changes in cue frequency and the post-session and functional surveys, calling the patient when these were insufficient. More frequent cues led to an easier programme and rarer cues to a harder one (a new template, or changed *R* and durations), while ‘the first training’, the first session of the day, was intended as the control; its most frequent template covered 70, 57, 68, 93, 83, and 97% of first sessions in P1–P6. Review dates, reasons, and the time spent on a review were not recorded; neither the adjustment rules nor any rule for stopping the training or for referral was written down in advance. A batch of scheduled sessions was created on each review day; 16 of the 28 intervals between a patient’s batches were 25–35 days. The physician’s interface shows per-step records and daily cue counts, which served, as intended, for the remote adjustment of the programme (consequence for the trend analysis: Section 2.7). For the analysed exercises, *R* changed only with the template: no execution exceeded the current *R*, and every one that fell short had reached the time limit. The purpose of the adjustment was a manageable load and sustained motivation.

Three of the 36 exercises performed were analysed: 180° abduction, 90° abduction, and the hand-to-mouth task. They were chosen because the hand-to-mouth task is functional, 180° abduction shows a wide range with combined deviations, and 90° abduction contrasts range demands; the time per repetition adds to the pace. Each patient did each exercise on 96–100% of active days. The catalogue gives one sentence each (‘abduct the arm to 180 degrees’, ‘abduct the arms to 90 degrees’, ‘bring the hand to the mouth’), without starting position, plane, pace, or arm. The instruction was to sit, to abduct in the frontal plane with the elbow extended, to follow the demonstration video, preferably at its pace, and to make the start gesture (Section 2.4) with the non-paretic arm. The arms were to take turns in all six patients, starting with the non-paretic arm; the non-paretic arm alone was allowed if the paretic arm could not perform, on the rationale of cross-education [49]. The 90° exercise, despite its plural, was designed for one arm at a time. Whether the paretic arm reached the target was not recorded.

The patient sees the mirrored image with the skeleton, a repetition counter, the remaining time, and a phase instruction; a cue is shown as red text only until the repetition is counted, and a demonstration video can precede each step.

An optional four-question post-session survey (exercises clear; everything accomplished; load tolerance; well-being) is visible to the physician. Its answers, from a third export (22 September 2026), are described per patient, without relation to the cue rates, with definitions written before they were seen. The physicians’ functional survey (arm pain, lifting the paretic arm, active abduction) is not part of the application, and its answers are not in the database.

### 2.4. Detection of Compensatory Patterns and Real-Time Cues

The application estimates the pose in each camera frame, checks for up to four compensatory patterns—lateral trunk lean, shoulder elevation, elbow flexion, and head tilt—by threshold rules on the landmarks and, when it detects one, shows the corresponding cue as text in real time (Table 2). The description is taken from the source code (front-end 1.9.2rc1, July 2026). The cue rules and their thresholds were not changed during the study.

#### 2.4.1. Pose Estimation

Pose estimation runs in the browser on the patient’s computer (MediaPipe Holistic JavaScript solution [50,51] with the full BlazePose model [52], built-in landmark smoothing, and minimum detection and tracking confidences of 0.5). No camera resolution is requested (library default 640 × 480 pixels), and the resolution delivered depends on the device; frames are processed one at a time, the next being requested when the previous has been processed, so the frame rate also depends on the device. Neither was recorded, and the application version, device model, operating system, and browser are not in the database. The rules use two-dimensional landmarks in image-normalised coordinates (*x* and *y* divided by the image width and height). There is no calibration step, aspect-ratio correction, body-size normalisation, visibility check, resting baseline, or minimum duration: a cue is decided on a single frame. The start gesture that begins a session (a hand to the knee, then the wrist above the shoulder) sets no threshold.

#### 2.4.2. Counting Repetitions

In abduction, a repetition is counted when the image-plane shoulder angle (between the shoulder–hip and shoulder–elbow segments) of either arm exceeds 168° in the 180° exercise or 78° in the 90° exercise, and the counter is re-armed below 30°. In the hand-to-mouth task, it is counted when a middle-finger landmark comes within 0.035 (normalised units) of the mouth midpoint while that arm’s elbow angle (the image-plane angle at the elbow between the elbow–shoulder and elbow–wrist segments) is below 50°, and the counter is re-armed above 100°. Either arm can score, and the side is not recorded.

A step ends at *R* repetitions or at its time limit. The whole-second step timer stops during the start gesture that begins a session, the demonstration video, pauses, and the help window.

#### 2.4.3. Cue Rules

The cues use three left–right asymmetries and, in abduction, the elbow angle: $d_{Y}=\left|y_{\mathrm{sh}}^{L}-y_{\mathrm{sh}}^{R}\right|$, the difference in shoulder height (fraction of the image height); $D=\left|\left|x_{\mathrm{sh}}^{L}-x_{m}|-|x_{\mathrm{sh}}^{R}-x_{m}\right|\right|$, the asymmetry of the horizontal distances from the shoulders to the mouth midpoint *x*_m_; and $E=\left|\left|x_{\mathrm{ear}}^{L}-x_{m}|-|x_{\mathrm{ear}}^{R}-x_{m}\right|\right|$, the same for the ears (fractions of the image width). At most one cue is selected per frame (Table 2), in the priority order trunk, shoulder, elbow, head. When *E* ≤ 0.02 and *D* lies in an intermediate band (the suppression band, rule 2), no cue is shown. The counts are therefore not independent: a frequent cue of higher priority, or a posture in the suppression band, lowers the others.

The shoulder cue is unsigned: it cannot detect elevation of both shoulders, and it responds to lateral trunk inclination and camera tilt. The trunk cue is a lateral shoulder asymmetry about the mouth; the hips are not used, and forward lean is invisible in a frontal view. The head cue requires a large ear-to-mouth asymmetry (*E* > 0.04), as a turn of the head can produce; in the technical check (Section 3.5), it almost never responded to head tilt, which often produced the trunk cue instead, and head rotation was not tested. The elbow cue fires for either arm, including the idle arm and the rest between repetitions, so it need not reflect the elbow during the raise. No rule targets trunk rotation or assistance from the non-paretic arm, and shoulder abduction enters only in the counting of repetitions. The thresholds are in image-normalised units, so the same posture gives different values at different distances from the camera, and *D*, *E*, and the angles depend on the aspect ratio of the image.

#### 2.4.4. Counting Cues

A cue type is counted at most once per repetition; several types can be counted in one repetition, and only per-step totals are stored. In abduction, the rules are evaluated from the return below 30° to the next count (the rest and the raise, not the lowering); in the hand-to-mouth task, in every frame. The probability of a count therefore rises with the time per repetition; its dependence on the frame rate differs between cues (in the technical check below, a lower frame rate made the elbow cue rarer and the trunk cue more frequent).

#### 2.4.5. Data That Leave the Device

Each step sends one record: the step number, the counted repetitions, the time spent, and the cue texts with their counts. The application records and transmits no frames, landmarks, or audio (Figure 1). The latency from camera frame to cue was not measured. Loss of pose detection, displacement of the camera, offline periods, and failed transmissions are not logged. Without a detected pose, no repetition is counted and no cue is shown, so a lost detection appears in the log, if at all, only as a longer time spent, a step that reached its time limit, or a step without cue records (Section 2.5 and Section 2.6); a step whose record did not reach the server is absent from the log. No alert is sent to the physician, who sees the records at the programme reviews (Section 2.3). The records are sent over HTTPS and stored on the server under the patient’s account; the patient’s and the physician’s interfaces require a login.

#### 2.4.6. Thresholds and Validation

The thresholds of the cue rules were set in advance on reference videos recorded by the physicians and were kept fixed while the patients used the application; they were not derived from published evidence or from the data of this study. Apart from the pose estimator, no classifier, machine-learning model, or individualised threshold is involved, and no rule or threshold was fitted, tuned, or selected on these data. The patients’ sessions also have no reference standard: no training session was recorded on video or observed by a clinician, in person or during the video calls (Section 2.3), and none of their repetitions was rated. A research detector built on the same approach—rules with fixed thresholds on joint angles and landmark distances—was validated in our previous study against clinicians’ annotations in healthy adults simulating compensations in one exercise (a seated bilateral arm raise to about 90°) [37,53]. The rules deployed here predate that detector and differ from it in thresholds, coordinate normalisation, the absence of a resting baseline, the temporal rule (a single-frame decision) and the arbitration (one cue per frame by priority); their accuracy in patients is unknown.

#### 2.4.7. Technical Check in Healthy Adults

The deployed 90° cue selection and repetition counting were replayed frame by frame, thresholds unchanged, on the landmarks of our previous study [37] (Section 3.5). There, 18 healthy adults, filmed frontally, performed 621 repetitions of seated bilateral straight-arm abduction to about 90° in six blocks each, one per condition: correct (177) or simulating elbow flexion, inter-limb asymmetry, shoulder elevation, trunk lean, or head tilt. Two physicians in physical and rehabilitation medicine, each with ten years’ experience and not involved in the sessions, labelled the repetitions from video [53], blind to the instructed condition, the system output, and each other’s labels (Cohen’s *κ* 0.96, 0.62, 0.73, 0.97, and 0.93, respectively; intra-rater reliability not assessed). Their agreed label was the clinical reference standard, with the 5–84 disagreements per pattern excluded, not adjudicated. Against either clinician alone, estimates other than counts changed by at most 0.022, except the shoulder cue’s negative predictive value, which fell to 0.75 against one clinician. A repetition was cued if the cue was counted between its start and its count at 78°, before the hold, whereas the clinicians judged the whole repetition, hold included; the application’s per-count attribution (adding the preceding rest) is also reported. All repetitions stayed in the denominators; those never reaching 78° were evaluated whole. The 95% confidence intervals come from 2000 bootstrap resamples of participants; predictive values from fewer than 20 cued repetitions are given as counts. The landmarks were MediaPipe PoseLandmarker output on 16:9 frames, smoothed by the recorder, whereas the application used Holistic on 4:3 images by default, so the replay was repeated on a 4:3 crop, unsmoothed and at 15 and 10 frames per second; Holistic was not emulated. The raise was bilateral; the patients’ exercise was designed for one arm at a time (Section 2.3). Neither the 180° rules (the count requires 168°, whereas the median peak shoulder angle in these recordings was 109°) nor the hand-to-mouth rules could be checked.

### 2.5. Data Source and Processing

The data are a snapshot of the production database of 17 September 2026 restricted to the six patients; it and two later exports, likewise restricted, were made by A.E.P. For each completed step (a step execution), it holds the step number, completed repetitions, time spent, completion time, and, per cue type that fired, the repetitions with that cue. Nothing is recorded between steps or outside sessions, so everyday activity does not enter the log. No video or landmarks are stored, so the counts cannot be recomputed. The clinical data of Table 1 were joined to the log by patient code; no other linkage was performed. Cue counts exist from 10 November 2024; earlier sessions only describe prior use (Table 3). All completed sessions were used, including those of courses later marked as deleted. The step-to-exercise mapping and *R* come from the template catalogue of the snapshot, which has no version history.

Of 132,096 step executions, 36,858 fell within the windows after removal of rest breaks, of one execution whose step is absent from its template, and of executions before 10 November 2024; 4013 were of the analysed exercises.

#### 2.5.1. Control Session

The control session is the first completed execution of an exercise per patient and calendar day (Moscow time), in any template, considering a one-step execution, not a session, and the first step if a template holds the exercise twice. The term comes from the programme (‘the first training’; Section 2.3); our definition coincides with it only on days on which the first session of the day contained the exercise. It needs no choice of template; the dominant template alone (SA2) and all executions of the day (SA3) are sensitivity analyses (Section 2.7). It gave 758, 759, and 760 control sessions for 180° abduction, 90° abduction, and the hand-to-mouth task (2277); 757, 759, and 759 (2275) remain for the cue rates.

#### 2.5.2. Control Sessions Without Cue Records

When no cue fires, an empty list is sent and no cue record is stored. A control session without cue records is counted as zero if all *R* repetitions were completed and another step that day had cue records; two failed the first condition (the time limit expired before any repetition was counted) and were excluded. In any control session without cue records, a failure to record cannot be told from a true absence of cues, so the share of such control sessions is reported (Table 4) and their exclusion is a sensitivity analysis (SA4; Section 2.7). Where this share reached 50% or more (the hand-to-mouth task in P4 and P5), the change was not categorised, because zero-filling and exclusion bias it in opposite directions.

### 2.6. Outcomes

#### 2.6.1. Primary Outcome: Rate of the Compensation Cues (Cue Rate)

For a control session with *R* prescribed repetitions and *n_c_* repetitions in which cue *c* was counted, the cue rate is *r_c_* = *n_c_*/*R*, between 0 and 1, because a cue type is counted at most once per repetition. All but five of the 2275 control sessions reached *R* (the five ended at the time limit; *R* remains the denominator). The range reached beyond the counting threshold and the duration of a pattern are not recorded. We report the share of control sessions with the cue and the mean cue rate (Table 4). The analysis centres on the leading cue of each exercise, chosen by frequency before the present analysis: the elbow cue (elbow flexion) in both abductions (the most frequent cue at 180°; at 90° chosen by analogy, before any model for this exercise was fitted) and the shoulder cue (shoulder elevation) in the hand-to-mouth task. These cues lack the highest priority, so other cues can partly suppress them (Section 2.4). As either arm can score (Section 2.4), each rate refers to both arms.

The cue rate also depends on the pace and the frame rate (Section 2.4). It was not adjusted because the frame rate was not recorded and the pace may change with training. Instead, the change in time per repetition is shown next to every cue-rate change (Figure 2; Table 5 and Table 6), and a decrease with faster repetitions is not read as a change in posture.

#### 2.6.2. Secondary Outcome

The time per repetition is the time spent on the step divided by the completed repetitions. The time spent is recorded in whole seconds and excludes stopped timer periods (Section 2.4), but includes idle time and the delay before the first repetition, which covers the loading of the pose model. The time per repetition describes the pace of a block, not movement duration or quality, and depends on *R* and the template. Control sessions ending at the time limit were excluded (7 of 2277).

#### 2.6.3. Training Volume and Regularity

A session is an assignment marked as completed, dated by its first step. A step is completed by the application’s count or time limit (Section 2.4); no clinician or relative confirmed completion. We report active days (with at least one completed exercise step) and their share of calendar days; completed sessions, in total and per active day; the median session duration (summed step times including breaks but not videos or pauses, so shorter than the wall-clock time); the recorded training time (sum of the step timers of completed sessions) and the repetitions counted by the application for either arm (Section 2.4); control sessions; gaps without exercise (the longest and those of at least 22 days); and prior use (Table 3).

The snapshot holds completed assignments only, so the schedule was taken from a second, unfiltered export of 21 September 2026, requested after the main results; its definitions preceded its analysis, except for the daily sum of the time limits and the contents of sessions started but not completed, which were added after the first results. A scheduled session is an assignment due within the window; it is completed, started but not completed (at least one step recorded), or not started; the log holds no reason for a session not completed or not started, nor for a day without training. Assignments that the physician deleted at a course replacement and that were not completed are excluded in the main variant and counted in a second. Deletion is marked per batch, without a time; as a check made after the analysis showed that 472 of the 522 such assignments had fallen due before the last completed session of their batch, that is, before withdrawal, the second variant is closer to the schedule actually in force. The patients were expected to complete the whole schedule, the recommended load. The relation of volume to the change in cue rates was not analysed.

### 2.7. Statistical Analysis

No sample size was calculated; the series comprises the six selected patients (Section 2.2). The patient is the unit of inference, and control sessions are repeated measurements within a patient. All analyses are per patient, without pooling, group-level tests or comparisons of levels; with six patients, a mixed-effects model cannot be estimated reliably. The group is summarised by the number of patients per category; as windows differ in length, these counts do not compare the size of change.

#### 2.7.1. Change in Cue Rate

For every patient, exercise, and cue, a binomial logit model [54], logit(*p*) = *α* + *βt*, was fitted to *r_c_* with weight *R*; *t* is the time from the start of the window in months (days/30.44). No term for the template or *R* was entered. The template was changed in response to the cues (Section 2.3), so it is not exogenous, and a term for it would absorb part of the change. The trend is therefore the trend under the programme as adjusted, not under a fixed task. SA2 keeps only the dominant template, whose first and last control sessions are themselves placed at reviews; it shows whether a category holds within one template and does not estimate a fixed-task trend.

Overdispersion and serial correlation were handled by a heteroskedasticity- and autocorrelation-consistent (HAC) covariance (Bartlett kernel, seven lags, small-sample correction *n*/(*n* − *k*)) [55]. Lags count control sessions, regardless of calendar gaps. A model required at least 20 control sessions and 20 repetitions each with and without the cue. The bandwidth, chosen after exploratory fits, and this rule were applied to all series; 3 and 14 lags were added after the final analysis. HAC intervals tend to be too narrow in short series (P4, 45–46 control sessions; sparse sensitivity analyses).

The main result (Table 5) is the model-based difference in fitted cue rate between the last and the first control session, in percentage points (pp), with a delta-method 95% confidence interval. The linear logit term summarises the direction; linearity was not tested, and the difference is taken from the fitted line at the window ends, in some series reaching two to three times the observed difference between the mean cue rates of the first and the last 30 days of the window (Figure 3). Its size is not interpreted beyond its category. The time unit (the control session), the window ends, and these 30-day blocks were fixed in the analysis plan (Section 2.1).

A series with *r_c_* ≥ 0.9 in over half its control sessions is at the ceiling, where an increase cannot be measured. A series with a fitted first rate below 10% is at the floor, where no marked change means only that a rare cue stayed rare; such series are not counted among patients without marked change (added after the final analysis; it changes no estimate or category).

The categories were defined before the final analysis. A decrease is a difference ≤ −10 pp with the upper confidence limit below zero; an increase is defined symmetrically; no marked change means the whole interval lies within ±10 pp; and the rest is undetermined. The threshold is one repetition in ten (*R* was 6–18): a convention without a clinical anchor, agreed with the treating physicians (alternatives: 5 and 15 pp). A series whose model-based and observed 30-day differences have opposite signs, either reaching 10 pp, is flagged and not described in words.

#### 2.7.2. Change in Time per Repetition

Series with at least 20 control sessions not ending at the time limit (Section 2.6) were modelled on the log scale with the same HAC covariance, giving the percentage change between the last and the first control session (95% Wald interval); the observed change is that between the first and last 30-day medians. The categories faster, slower, no marked change, and undetermined use 15% (alternatives: 10% and 20%), set after the medians had been computed and before any time model was fitted, and agreed with the treating physicians; the sign flag applies as above. SA4 does not apply, because the time record does not depend on the cue records.

#### 2.7.3. Sensitivity Analyses and Stability

Each sensitivity analysis changes one element. Time is counted from the start of the main window, and the difference is taken between the first and last control session of the analysis, so agreement concerns direction and threshold, not size.

- SA1: Window cut at the end of month 6 (applicable to P3 and P5).
- SA2: Dominant template only (most control sessions), with the control session redefined within it (applicable where that template covers less than 95% of control sessions).
- SA3: All executions of the day combined (4013 executions).
- SA4: Control sessions without cue records excluded (applicable where they are at least 5%).
- SA5: First 14 days excluded (always applicable); planned as familiarisation, this has that meaning only for P5.
- SA6: Control sessions before 15 February 2025, where present, excluded; a stored field changed that day, showing a server update, and the version is not stored.
- SA7: Series continued to 31 August 2026 (P1, P2, P5 and P6; up to about 19 months).

A category is stable if it is the same in the main analysis and in every applicable analysis among SA1, SA2 and SA4–SA7; if one of them cannot be evaluated, the category is not stable. Only stable categories are described in words; others are reported as depending on the definitions that change them. By a convention of the plan, SA3 is outside the rule; the stable categories it changes are counted in Section 3.3.

The intervals are two-sided 95% intervals without adjustment for multiplicity. Each category thus acts as an unadjusted 5% test, and some will arise by chance. Stability does not protect against this, because the sensitivity analyses reuse most control sessions. The categories are descriptive labels; the intervals express precision.

### 2.8. Reproducibility

One script produces all tables and figures of the case series from the database snapshot, whose checksum is verified first; two consecutive runs give identical output. It uses Python 3.9, pandas 2.3, and statsmodels 0.14 [56] and is available from the corresponding author upon reasonable request, as is the replay script for the technical check (Section 2.4).

## 3. Results

### 3.1. Home Training over Months: Volume and Adherence

Over windows of 3.8–9.9 months, the six patients trained at home for 46–222 days (33–96% of the calendar days; more than two-thirds in four patients) and completed 2428 sessions (122–786 per patient; 1.8–5.3 per active day; Table 3). The median recorded duration of a session was 5.5–17.9 min, and the median recorded training time per active day was 18–68 min. Per patient, 19–167 h of training were recorded and 19,379–114,571 repetitions counted (Table 3). Gaps of at least 22 days occurred once each in P3, P4, and P5. Sessions were scheduled for 92–100% of the calendar days, a median of two to six per day (four to six counting the assignments that were deleted and not completed). Their time limits added up to a median of 1.8–4.0 h per day. The patients completed 13–93% of the scheduled sessions, or 13–90% counting the deleted assignments, the closer variant (Section 2.6; per patient in Table 3). Five of the six completed less than half in both variants. Sessions stayed scheduled through the gaps of P3 and P4; the gap of P5 was a gap in the schedule. After the window, 0–67% were completed. These shares are not compared with the window, which for four patients ends at a data cut-off.

In P5 a further 20% of the scheduled sessions were started and not completed (at most 2% in the others); 258 of these 273 sessions (deleted assignments counted) stopped immediately before one exercise, both hands to the knees. In P1 this exercise was recorded 363 times without a counted repetition and took 11% of the recorded training time. Some patients found this exercise difficult and could abandon a session there, but the log holds no reason for abandoning, so a movement that was not performed cannot be distinguished from one that was not counted. Steps of sessions not completed are outside Table 3; they hold 17% of the repetitions and about 15% of the recorded training time of P5, and at most 5% in the others. The three analysed exercises made up 10–23% of the repetitions. Five patients had used the application before the window (84–676 completed sessions); four had 156–292 control sessions per exercise after it, up to 31 August 2026. Five of the six were still training in mid-2026 (last completed sessions dating from 5 June to 31 August 2026, the end of the analysed data).

The post-session survey was answered after 200 of the 202 completed sessions of P2 and 783 of the 786 of P6, after 2% of those of P5, after one of P3, and after none of P1 and P4. P6 gave the same four favourable answers every time. P2 answered that everything was accomplished only partly after 99% of the sessions, that the load was tolerated well after 74% and satisfactorily after 26%, and that she felt well after 56% and satisfactorily after 44%. Within the windows, no patient answered that the load was tolerated poorly or that he or she felt poorly.

### 3.2. Compensation Cues Shown by the System

In 180° abduction, the system showed the elbow cue in 91–100% of the control sessions for every patient (mean cue rate 46–90%; Table 4); in P2, P5, and P6, it was shown in every repetition in 15–24% of the control sessions, and the series of P5 met the ceiling criterion (Section 2.7). In the hand-to-mouth task, the system most often showed the shoulder cue, shown in 27–99% of the control sessions (mean cue rate 2–57%). In these two exercises, the other cues were shown less often than the leading cue in every patient. In 90° abduction the elbow cue was shown in 44–99% of the control sessions (mean cue rate 4–54%) but was not always the most frequent cue: the shoulder cue was more frequent in P1 (mean cue rate 26% against 19%) and P6 (28% against 13%), and in P2 the head cue (16% against 13%) and, marginally, the shoulder cue (13.4% against 13.0%).

Of the 2277 control sessions, 275 had no cue records, and two of these were excluded (Section 2.5). The share without cue records was below 3% in most series and reached 71% (Table 4); within series it was not constant over time (in the 90° abduction series of P2, 36% in the first 30 days and 14–19% thereafter).

### 3.3. Long-Term Dynamics of the Leading Cues Within Patients

Per-patient models described the rate of the leading cue over each window (Figure 2A and Figure 3; Table 5). Of the 18 series of leading cues (six patients, three exercises), 17 could be modelled and 16 could be categorised. Four, all of the elbow cues in P1 and P6, were stable: their category held in every applicable sensitivity analysis, including the continuation of the series to 31 August 2026 (SA7; Section 2.7). In P6 the elbow cue rate decreased in 180° abduction (−61.7 pp, 95% confidence interval (CI) −76.1 to −47.3; observed means 87.5% in the first 30 days and 33.6% in the last 30), whereas in 90° abduction it showed no marked change (2.2 pp, −2.3 to 6.6; 13.7% and 14.5%). In P1 it decreased in 90° abduction (−32.3 pp, −50.6 to −13.9; 21.4% and 10.7%) but increased in 180° abduction (74.7 pp, 59.2 to 90.2; 28.6% and 89.3%). The direction thus differed between the two patients in the same exercise and between the two exercises in P1. The three directional changes also held with thresholds of 5 and 15 pp (the series without marked change only with 15 pp), and all four with all executions of the day (SA3) and with HAC bandwidths of 3 and 14 lags. In these four series, at most two of 119–149 control sessions lack cue records (Table 4), so no assumption about those sessions, whether counted as zeros (the main analysis), excluded, or counted as showing the cue in every repetition, can change the category.

The shoulder cue of P4 in the hand-to-mouth task had too few events for a model, and that of P5 was not categorised, because 68% of its control sessions have no cue records. The other twelve categorised series, including all four categorised hand-to-mouth series, are not described in words: in ten the category changed in at least one applicable sensitivity analysis (SA6 in six; SA1, SA5 and SA7 in four each; SA4 in two), and in the two abduction series of P4, SA6 could not be evaluated because too few control sessions remained (estimates in Table 5). In eight of them, the dominant template covered less than 95% of the control sessions, and within it (SA2) the category changed in five.

The cue rate depends on the pace (Section 2.6). In the 90° series of P1, the time per repetition shortened (−24.1%, 95% CI −34.9 to −11.5; Table 6). This change was not stable: in the series continued after the window (SA7), the time lengthened (34.2%, 16.9 to 54.0), while the cue rate remained decreased (−20.6 pp, −29.6 to −11.6). In the two stable series of 180° abduction, the change in time was undetermined (P1: 0.2%, −20.6 to 26.5; P6: −9.1%, −19.7 to 2.8). A contribution of the pace can therefore be neither established nor excluded in these series, and the trajectories are reported as changes in the cue rate, not as changes in posture.

In 180° abduction of P1, the elbow cue rose while the shoulder cue, which has priority over it within a frame (Section 2.4), fell (observed means of the first and the last 30 days: 52.7% and 8.0%). A shoulder cue shown less often leaves more frames for the elbow cue, so part of the rise may come from the selection rule and not from the position of the elbow; its size cannot be determined from per-step counts. In P6 the shoulder cue rose (0.9% and 10.7%), which by the same rule acts towards the observed fall of the elbow cue. The shoulder cue can suppress the elbow cue only in repetitions in which it is itself counted, so this rise can account for at most 10.7 of the 53.9 pp fall, which amounts to about a fifth.

Over all cues (four in each abduction, three in the hand-to-mouth task, where the elbow rule is not applied; 66 series), 53 met the minimum-data rule and 51 were categorised: 12 as a decrease, 10 as an increase, 11 as no marked change, and 18 as undetermined. Seventeen were stable: four decreases, two increases, six without marked change and five undetermined. Ten of the 11 series without marked change, including five of the six stable ones, were flagged as at the floor (Section 2.7). Thresholds of 5 and 15 pp instead of 10 pp changed 11 and 16 of the 51 categories. HAC bandwidths of three and 14 lags, instead of seven, changed one and three categories, none of them stable. The only leading series among them (P2, elbow cue in 90° abduction) already depended on the definitions. All executions of a day instead of the first (SA3) changed 13 of the 51 categories, including two stable ones: in P6, the shoulder cue in 90° abduction and the head cue in the hand-to-mouth task. One series was flagged because the model and the observed 30-day means disagreed in sign (P1, shoulder cue in 90° abduction).

### 3.4. Time per Repetition

The median time per repetition in the first 30 days was 2.5–4.6 s in 180° abduction, 1.6–2.9 s in 90° abduction, and 1.6–4.4 s in the hand-to-mouth task. All 18 series were modelled (Figure 2B, Table 6): six were faster, three were slower, and nine were undetermined. Three categories were stable. P5 became faster in the hand-to-mouth task (−39.4%, 95% CI −50.7 to −25.5; −28.5% within the dominant template), and the change was undetermined in P3 in the hand-to-mouth task and in P6 in 180° abduction. P4 became slower in all three exercises (43–48%), but SA6 could not be evaluated in this patient, so these categories are not regarded as stable. In the other 12 series, the category depended on at least one sensitivity analysis, most often SA7. Thresholds of 10% and 20% instead of 15% changed none and five of the 18 categories (one stable: P6, 180° abduction, no marked change at 20%). A HAC bandwidth of three lags changed one of the 18 categories (P4, hand-to-mouth task; not stable), and one of 14 lags changed none.

### 3.5. Technical Check of the 90° Rules in Healthy Adults

The 90° rules were specific (0.90–1.00) but detected only a minority of the simulated compensations (Table 7; below, a second value in parentheses is for the 4:3 crop). They counted 616 (617) of the 621 repetitions and cued one of the 177 correct ones at both aspect ratios. The elbow cue was shown in 45% (34%) of the 82 flexion repetitions; 15 (21) of the 45 (54) missed repetitions were not cued because, up to the count, the elbow angle fell below 150° only in suppression-band frames. Of the elbow-cued repetitions with an agreed label, 37 of 41 (28 of 29) were rated as flexion, or 35 of 48 (27 of 31) under the per-count attribution. In the 17 participants whose flexion series was rated as flexion in every agreed repetition, their elbow cue rate under the per-count attribution ranged from 0 to 100% (median 20%) at both aspect ratios. The trunk cue was shown in 44% (56%) of the trunk-lean repetitions and, at least as often, in 44% (62%) of the head-tilt repetitions; the head cue was shown in none (6%) of the latter. The shoulder cue was shown in none of the 107 shoulder-elevation repetitions: the participants raised both shoulders, which *d_Y_* does not register. Of the 621 repetitions, the aspect ratio alone changed the number with the elbow cue from 42 to 29 and with the trunk cue from 73 to 101. Lowering the frame rate changed the sensitivities by up to 0.12, and undoing the smoothing cued six (one) of the 177 correct repetitions; neither changed these patterns.

## 4. Discussion

### 4.1. Principal Findings

Over windows of 3.8–9.9 months, the application monitored algorithm-detected compensation cues in 2277 control sessions of six patients who trained at home without a clinician present. The cues shown most often were the elbow cue in 180° abduction and the shoulder cue in the hand-to-mouth task (Section 3.2). Modelled within each patient, the rate of the leading cue followed individual trajectories. Four of the 16 categorised trajectories kept their category in every applicable sensitivity analysis, all for elbow flexion: its cue rate decreased in P6 at 180° and in P1 at 90°, increased in P1 at 180°, and showed no marked change in P6 at 90° (Section 3.3). The direction thus differed between patients and, in P1, between the two abductions, which a single measurement could not have shown. The other twelve depended on, or could not be tested against, analytical choices that log-based monitoring usually leaves implicit, such as the templates included, missing cue records, early sessions, sessions before a server update, and the end of the window. This is a result in its own right: such trajectories are informative only when these choices are fixed in advance and reported with sensitivity analyses.

Several features of the setting can explain why the trajectories did not share one direction. The physician made the programme easier when cues were frequent and harder when they were rare (Section 2.3); a programme titrated in this way tends to hold the cue rate steady, and a change in template can move it either way. The elbow cue at 180° was near its ceiling (Section 3.2); five patients had used the application for 1.4–5.9 months before the window, the analysed exercises made up 10–23% of the repetitions, and no baseline strength grade exceeded 3 (median ARAT score 8). The trajectories therefore describe the cues shown under an individually adjusted programme, not the response to a fixed task.

### 4.2. Interpretation of the Cues and Their Trajectories

The cue rate records what the rules detect. A cue signals that a threshold was crossed; whether the pattern was a harmful compensation or a task-appropriate adjustment, the rules cannot tell. Besides the posture, the rate reflects the pace, because the rules are evaluated frame by frame until a repetition is counted; the selection of one cue per frame by priority; and the patient’s response to the cue, which is part of the intervention (Section 2.4). A trajectory is therefore best read together with the time per repetition and the other cues, and never as a clinical course: the baseline scales were not repeated, so a fall in the cue rate is no evidence of improvement, nor is a rise evidence of deterioration. The four stable trajectories arose within one control task as recorded: all abduction control sessions of P1, and 97–98% of those of P6, came from one template, and hence, by the snapshot catalogue, with one prescribed number of repetitions (the catalogue keeps no template history); they therefore do not follow from a switch to another template, although the rest of the programme was adjusted around them. In P6 at 180° the rise in the shoulder cue can account for at most a fifth of the fall in the elbow cue (Section 3.3). The rest may reflect the posture of the performing or the idle arm, the pace, whose contribution was neither established nor excluded, or the camera position; per-step counts cannot distinguish these, but stored landmarks could (Section 4.5). More generally, a fall in the elbow cue rate can come from less frequent flexion, from flexion that no longer crosses 150°, or from a posture or camera position that keeps *D* in the suppression band. Conversely, no marked change, as in P6 at 90°, does not exclude a change in flexion. In healthy adults raising both arms to about 90°, the elbow cue rate in series rated as flexion throughout ranged between persons from 0 to 100% (Section 3.5), so its level cannot be compared between patients, and whether it follows a change in flexion within a patient was not tested.

The effect of the unrecorded conditions (Section 2.3) on the two-dimensional rules follows qualitatively from their definition (Section 2.4). The asymmetries *d_Y_*, *D*, and *E* are in image-normalised units, so a greater distance from the camera lowers them and, with them, the rates of the shoulder, trunk and head cues (and, through cue priority or the suppression band, can raise or lower the elbow cue). The elbow angle, taken in the image plane, changes with the viewpoint and the aspect ratio, which also rescales *D* and *E*; in healthy adults, a 4:3 instead of a 16:9 image alone lowered the number of repetitions with the elbow cue and raised that with the trunk cue (Section 3.5). The frame rate changes the number of frames evaluated; in healthy adults, lowering it changed the sensitivities by up to 0.12 (Section 3.5). Day-to-day variation in camera placement thus adds noise, and a lasting change in placement, resolution, or frame rate can shift the cue rates of all exercises from that date. Without a record of the conditions, such a shift cannot be told from a change in posture, and a linear trend fitted across it would register a change. Lighting and clothing act on landmark detection, and furniture on the resting posture of the idle arm, to which the elbow rule also responds (Section 2.4); without stored frames or landmarks (Section 2.5), their effect on the cue rates cannot be analysed.

Elbow flexion, whose cue was the most frequent in 180° abduction, accompanies shoulder abduction in the flexor synergy after stroke [57], and in every patient its cue was shown more often at 180° than at 90°. That study, however, kept the shoulder at 90° and varied only the load, so it does not predict this difference, and two properties of the system favour higher rates at 180°: a repetition took longer (Section 3.4), so more frames were evaluated, and the elbow angle, computed in the image plane, is distorted when the raised arm leaves that plane. As the elbow rule responds to either arm and is evaluated from the moment the arms are down until a repetition is counted (Section 2.4), an idle elbow resting in flexion, for example with the hand on the thigh, satisfies it on its own, and flexion during a paretic raise that falls short of the target is credited to the next counted repetition. Rates near 100% can thus arise from the rule alone; in P5, who had no increase in tone and a strength grade of 1, the mean elbow cue rate was 90% at 180° and 54% at 90° (Table 4). In P1, the elbow cue rate changed in opposite directions in the two abductions, so a trajectory belongs to a patient and an exercise, not to the patient alone.

The arms were to take turns, and the performing arm is not recorded (Section 2.3 and Section 2.4), so at most about half of the counted repetitions can have been performed by the paretic arm, and each trajectory is that of both arms: a change in it may come from the paretic arm, from the non-paretic arm, or from a change in which arm’s repetitions reach the target and are counted. The direction of a trajectory therefore cannot be assigned to the paretic arm. In P5 (ARAT 0), the series cannot describe the paretic arm: with grade 1 strength it could hardly reach any of the three targets against gravity, so the counted repetitions were probably those of the non-paretic arm, and the cues came from whichever arm met a rule. A relative read the cues aloud, so the feedback arrived later and through another person, and the baseline assessment was made 356 days before the window. The series of P5, including the ceiling at 180° and the stable shortening of the time per repetition in the hand-to-mouth task, describes how the rules behaved under these conditions. In P1 (ARAT 6) and P6 (ARAT 2), whose series gave the four stable trajectories, how often the paretic arm reached the target, and whether this changed, is unknown.

### 4.3. Adherence and Training Volume

Training continued over months: the patients trained on 33–96% of the calendar days, more than two-thirds in four of them, with 1.8–5.3 completed sessions per active day (Section 3.1). The schedule was set high (a median of two to six sessions a day, whose time limits added up to 1.8–4.0 h): five patients completed less than half of it and one 90% (Section 2.6 and Section 3.1). Completion also reflects the fit between a template and the patient: most unfinished sessions of P5 stopped before the both-hands-to-knees exercise, which in P1 used up recorded time without a counted repetition; the templates were not adjusted for it. We do not compare the volume with programmes in the literature, because the content of the sessions is documented only at the level of templates, and we did not relate it to the cue-rate trajectories: with six patients, windows of different length, and a programme adjusted to the cues, such an analysis could not be interpreted.

### 4.4. Comparison with Other Work

Accelerometry provides long records of the amount of arm use but does not capture movement quality directly [58]. Laboratory longitudinal studies confirm the value of serial kinematic assessment but require a specialised environment [59]. In multiple sclerosis, pose estimation applied to videos of self-care tasks recorded at home by patients captured changes in dexterity over six months [60]. After stroke, pose estimation from a single consumer camera has analysed upper-limb movements in one session or a few [34,35,61]; in a 2022 review, only one camera-based compensation study took place in patients’ homes [20], and a home depth-camera programme lasted six weeks [30]. The present series extends camera-based monitoring of algorithm-detected compensation cues to months of home training, several sessions a day, with only per-step counts leaving the patient’s computer. As webcam classifiers of shoulder compensation generalised poorly to unseen patients [35], following each patient against the same patient’s earlier sessions, as here, is a natural design.

### 4.5. Implications for Remote Monitoring and Next Steps

Routinely collected feedback counts allowed the cues shown to each patient to be followed for months without video leaving the home, and the same counts served the physician for the remote adjustment of the programme (Section 2.3). Before such monitoring can serve as a measurement of compensatory movement, the detection rules need validation against clinical ratings in patients, for every exercise in which they are used; rules evaluated independently of one another, as in the detector validated in healthy adults [37], would also remove the coupling through cue priority (Section 4.2). The system should record the performing arm, the application version, the frame rate and image resolution, the camera position and distance, the time at which an assignment is withdrawn, the number of sessions expected on a day, and the reason a session is abandoned. Where consent permits, body landmarks should be kept, so that the counts can be recomputed when the rules change; they need far less storage than video and show neither the patient’s face nor the home. Repeated clinical assessment within a controlled design would then show whether the individual trajectories add information to a clinical assessment and whether they relate to recovery.

### 4.6. Limitations

Outcome: There is no reference standard for the patients’ sessions, so the deployed rules have not been validated in patients (Section 2.4): the cue rate is not a validated measure of compensatory movement, and its sensitivity, specificity and share of false cues in patients cannot be estimated. In healthy adults simulating compensations in a bilateral 90° raise, the 90° rules were specific but detected under half of the elbow flexion and about half of the trunk lean, reported head tilt, if at all, mostly as trunk lean, and did not respond to elevation of both shoulders (Section 3.5); the 180° and hand-to-mouth rules were not checked. No clinician compared the cues with their own assessment of the patients, and the video calls were not recorded (Section 2.4). Without video or landmarks, the counts cannot be recomputed (Section 2.5). At the ceiling (P5 at 180°), an increase cannot be measured. No trajectory, and no session without cues, can be assigned to the paretic arm (Section 4.2). A change in camera placement, resolution, or frame rate cannot be told from a change in posture (Section 2.3 and Section 4.2). The time per repetition includes idle time.

Data: A server update of unknown version fell within the windows (15 February 2025; SA6); the cue rules and their thresholds were not changed. A missing cue record cannot be distinguished from a true absence of cues; its share changed over time (Section 3.2), so counting such sessions as zeros can by itself produce a trend. The schedule comes from a later export without deletion times, and a rescheduled session cannot be recognised, so completion is bounded, not estimated (Section 2.6).

Design: The six patients were heterogeneous (ARAT 0–32) and were selected for long and regular series from the 18 who trained at home (Section 2.2); the selection favoured a working home set-up, not arm function, and whether it favoured digital experience is unknown. Feasibility is thus shown only for patients who maintained long, regular home training, and the selection, by physicians who had seen the counts, may also bias the trajectories. With no control group, one centre, one application and one rule set, the series does not generalise to other patients, centres or applications. The windows were fixed before this analysis: four end at a data cut-off, and five begin after 1.4–5.9 months of use (Section 2.2), so early change is not observed, and the first control session is not a baseline. A trend may also reflect spontaneous recovery (P2 was late subacute for about the first five weeks), concomitant rehabilitation, medication or admissions (no data), the training volume (Section 4.3), fatigue or pain (no data after discharge), a change in strategy (which arm performed or how the cue was answered), familiarity with the counting rules or changes in camera position. Days without training are not missing at random; their reasons, clinical events, and device or transmission failures are not logged (Section 2.4 and Section 2.6). As the programme was adjusted in response to the cues (Section 2.3), no analysis can recover the trend under a fixed task (Section 2.7). Nothing was blinded: the patients saw the cues and the physicians saw the counts. Adverse reactions were ascertained only through the support service and the post-session survey, which does not ask about falls, pain or fatigue (Section 2.2 and Section 2.3).

Analysis: Only three of the 36 exercises were analysed. The analysis plan was not pre-registered and was extended after preliminary results (Section 2.1); the thresholds are conventions agreed with the treating physicians. The categories behave like unadjusted tests, and their stability does not protect against chance. HAC intervals tend to be too narrow in short series, their lags ignore calendar gaps, and the linear trend on the logit scale gives only a direction (Section 2.7).

## 5. Conclusions

Long-term markerless home monitoring of algorithm-detected compensation cues, not of validated compensatory movement patterns, was feasible with a webcam application across 2277 control sessions in six patients with post-stroke hemiparesis selected for long, regular training records. The most frequent cues were the elbow cue in 180° abduction and the shoulder cue in the hand-to-mouth task. Modelled within each patient, the rate of the leading cue followed individual trajectories, but only four of the 16 categorised trajectories (all elbow cue) held in every applicable sensitivity analysis. These four show the potential of such monitoring; the other twelve show how far the trajectories still depend on the analytical definitions. All are exploratory observations. The rules are unvalidated in patients, and in healthy adults, the 90° rules, the only ones checked, detected a minority of simulated compensations; either arm may produce cues, recording conditions were not logged, and clinical measures were obtained at baseline only, so a change in cue rate cannot be read as a change in compensatory movement or as clinical improvement or deterioration. Validation of the rules in patients, logging of the performing arm and the recording conditions, and controlled studies with repeated clinical assessment are the next steps before such trajectories can be related to recovery.

## Figures and Tables

**Figure 1:**
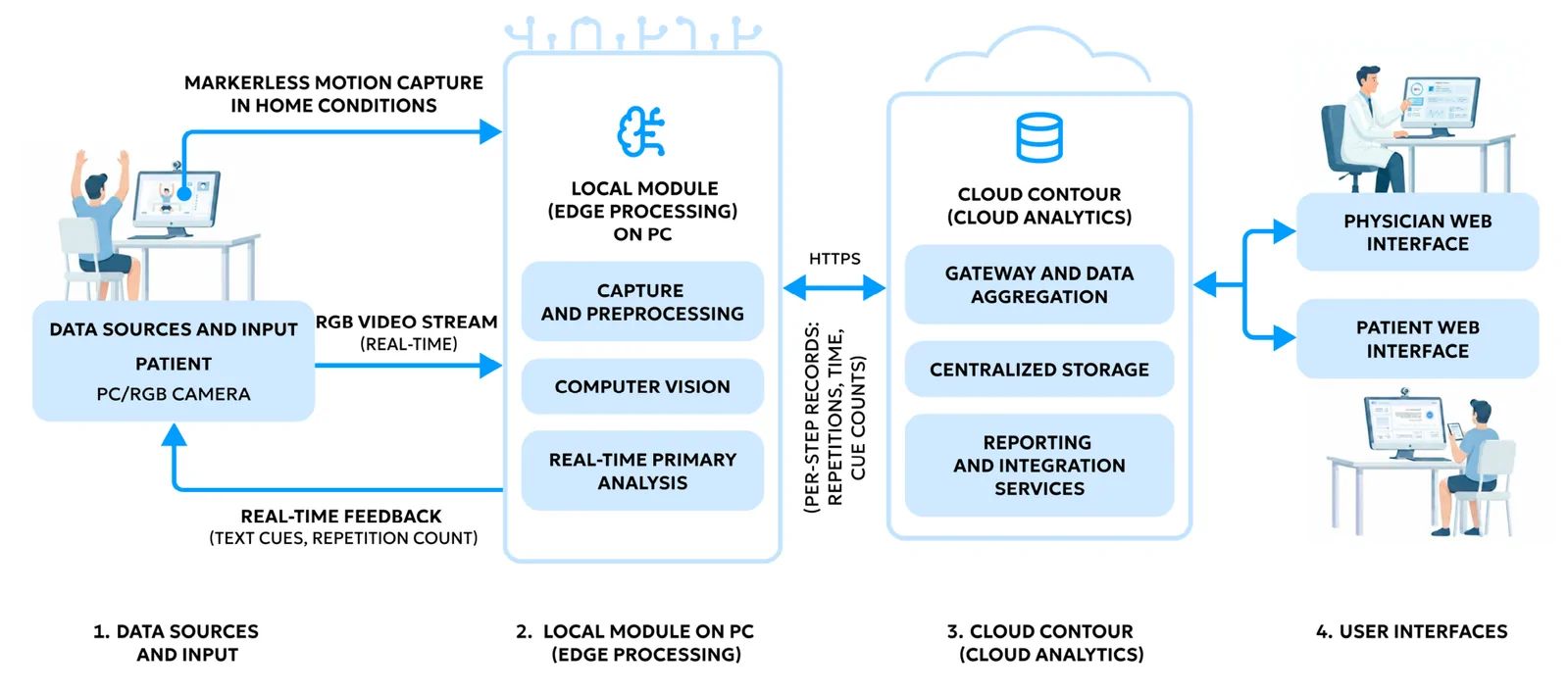
Architecture of the home-training system. (**1**) The patient exercises in front of the camera of a personal computer. (**2**) The local module runs in the browser on that computer: it receives the camera frames, estimates the pose, counts the repetitions, selects the cues and gives the feedback; the camera frames and the body landmarks do not leave the computer. (**3**) Only per-step records (repetition counts, time spent and cue counts) are sent to the server over HTTPS and stored. (**4**) The web interfaces show these records to the physician and the assigned sessions to the patient.

**Figure 2:**
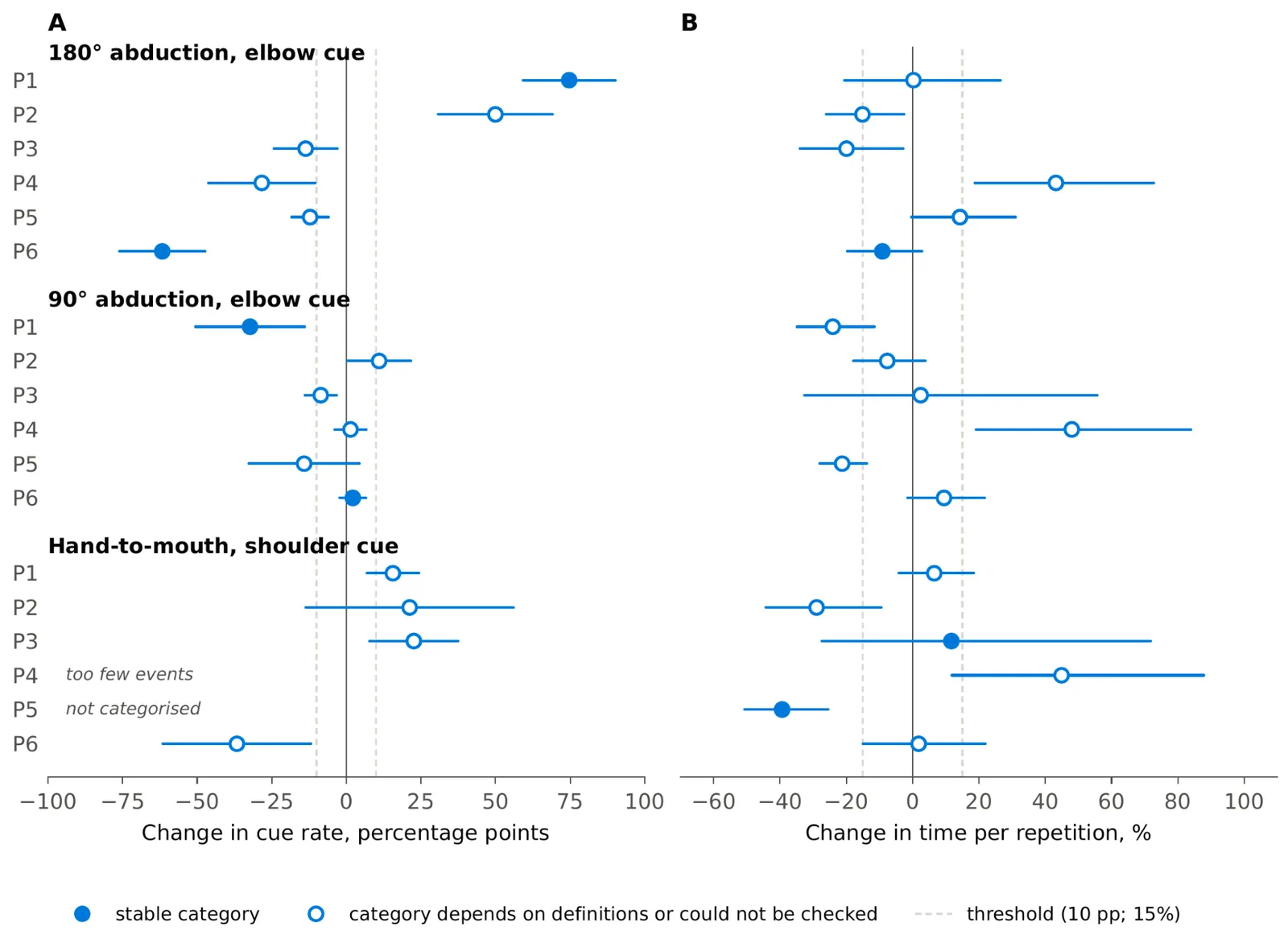
Model-based within-patient change between the first and the last control session of the observation window, with 95% confidence intervals. (**A**) Rate of the leading cue, percentage points. (**B**) Time per repetition, %. Filled markers are stable categories (Section 2.7); open markers are series whose category changes in at least one applicable sensitivity analysis or for which a sensitivity analysis could not be evaluated. Dashed lines mark the thresholds of 10 percentage points and 15%. For two series of leading cues no estimate is plotted: one had too few events for a model, and one was not categorised because more than half of its control sessions have no cue records.

**Figure 3:**
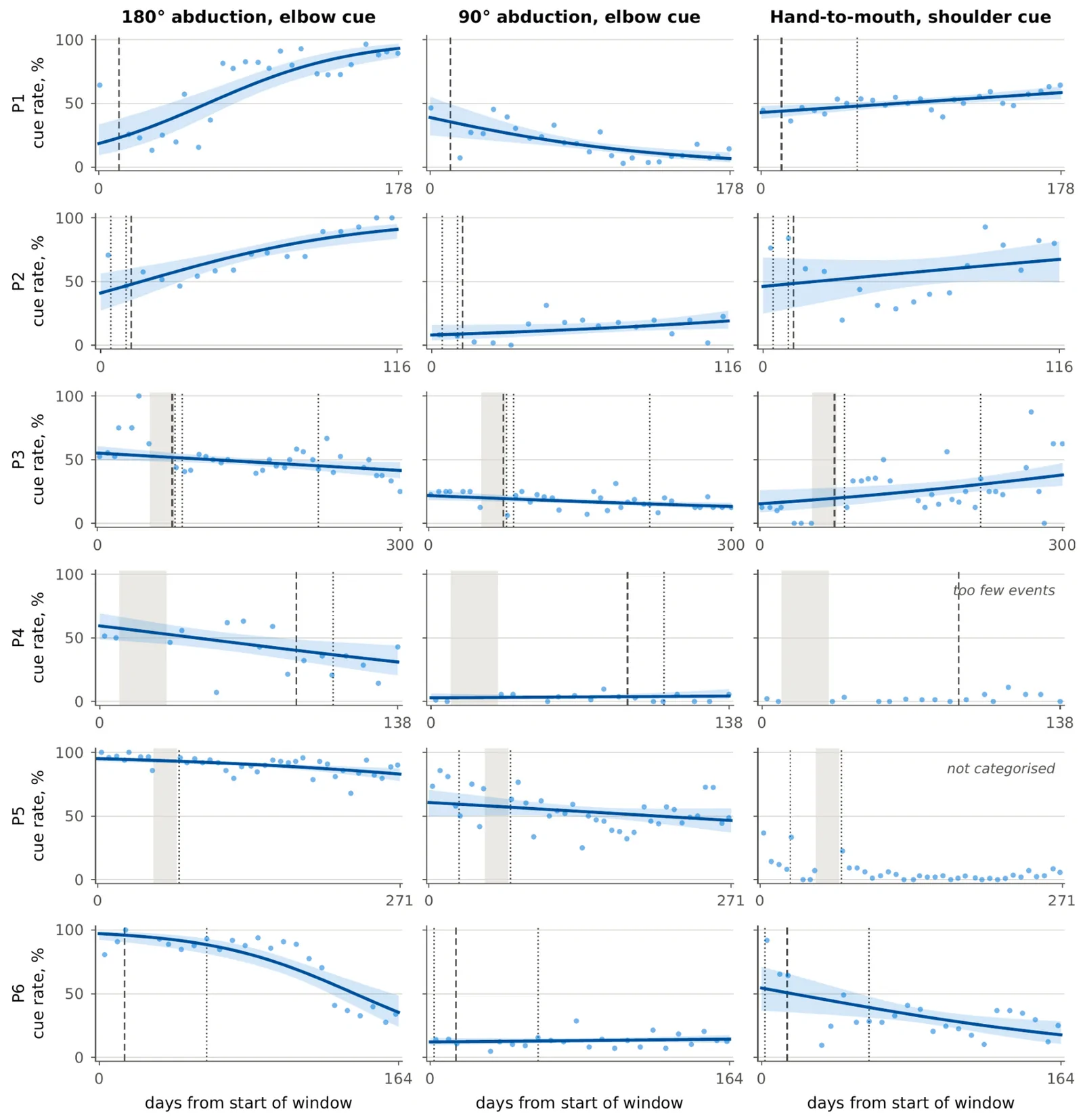
Rate of the leading cue within the observation window for the six patients (rows) and the three exercises (columns). Dots are means of the control sessions of one week; the curves are the fitted models with 95% confidence bands. The horizontal axis ends at the last day of each patient’s window. Dotted vertical lines mark the first control session from a template new to that series (templates were changed at programme reviews and alternated within days; Section 2.3); shaded bands are the gaps of at least 22 days (Table 3); the dashed line in P1–P4 and P6 marks 15 February 2025, the server update (SA6; Section 2.7); P5’s window begins after that date. A week without a dot had no control session. No curve is drawn for the series with too few events and for the series that was not categorised.

**Table 1:** Patient characteristics at baseline (at discharge from hospital).

							Shoulder/Elbow/Wrist				d440	
ID	Sex	Age	Side	Phase	Lesion	Months	Strength	Tone	ARAT	mRS	Perf.	Cap.
P1	F	54	R	chronic	left MCA	16	3/3/0	2/2/2	6	3	4	4
P2	F	61	L	late subacute	right MCA	3	3/2/2	2/2/2	10	3	3	3
P3	M	57	L	late subacute	right MCA	5	3/3/2	0/0/1 ^a^	32	3	2 ^b^	4 ^b^
P4	M	40	L	chronic	VB	11	3/3/2	2/2/3	26	3	3	3
P5	M	54	R	chronic	left MCA	12	1/1/1	0/0/0	0	4	4 ^c^	4 ^c^
P6	M	65	R	chronic	left MCA	9	3/3/2	2/2/2	2	3	4	4

Side—side of the hemiparesis (R, right; L, left); Phase—phase after stroke by the definitions of [41] (early subacute: 7 days to 3 months; late subacute: 3 to 6 months; chronic: more than 6 months); P2 is at the boundary at discharge; MCA—middle cerebral artery; VB—vertebrobasilar territory; Months—months from stroke to discharge (for the time since stroke and the phase on the first day of the observation window, see Section 2.2); Strength—Medical Research Council scale for shoulder abduction, elbow flexion and wrist extension (0–5, higher = stronger); Tone—Modified Ashworth Scale for the same levels (0–4, higher = greater tone); ARAT—Action Research Arm Test (0–57, higher = better function); mRS—modified Rankin Scale (0–5, higher = greater disability); d440—ICF category ‘fine hand use’, performance and capacity qualifiers (0–4, higher = greater difficulty). No patient reported pain (the assessment sheet recorded pain for P6 in error). ^a^ Empty cells of the assessment sheet, meaning no impairment. ^b^ Taken from the rehabilitation diagnosis in ICF categories; the values are printed as recorded. ^c^ The assessment sheet coded 0 and 0 in error; the values printed are the treating physicians’ correction.

**Table 2:** Cue selection in one frame, as implemented in the code snapshot of July 2026 (effective inequalities after combining the constants in the code). Rules are applied from top to bottom; the first that holds determines the cue. The cue texts are shown to the patient in Russian and are given here in English translation.

Rule	Condition and Cue	Abduction 180°/90°	Hand-to-Mouth
1	*E* ≤ 0.02 and *D* ≥ *T_D_*: trunk cue (“try not to lean”)	*T_D_* = 0.05	*T_D_* = 0.058
2	*E* ≤ 0.02 and *T*_0_ < *D* < *T_D_*: no cue	*T*_0_ = 0.01	*T*_0_ = 0.018
3	*d_Y_* > *T*_sh_: shoulder cue (“try not to raise the shoulder”)	*T*_sh_ = 0.07/0.041	*T*_sh_ = 0.026
4	elbow angle of either arm < 150°: elbow cue (“try not to bend the arms”)	applied	not applied
5	*E* > 0.04: head cue (“try not to tilt the head”)	applied	applied

**Table 3:** Training volume and regularity in the observation windows (application log).

	P1	P2	P3	P4	P5	P6
Window, days (months)	179 (5.8)	117 (3.8)	301 (9.9)	139 (4.5)	272 (8.9)	165 (5.4)
Active days (% of calendar days)	123 (68.7)	112 (95.7)	120 (39.9)	46 (33.1)	222 (81.6)	149 (90.3)
Completed sessions (per active day)	432 (3.51)	202 (1.80)	215 (1.79)	122 (2.65)	671 (3.02)	786 (5.28)
Median session duration, min	13.1	10.1	17.9	8.8	5.5	13.0
Recorded training time, h	95.1	34.7	67.4	19.3	72.0	166.6
Counted repetitions, all exercises (analysed exercises)	52,969 (7915)	42,137 (7138)	40,360 (4708)	19,379 (4412)	68,333 (10,440)	114,571 (11,138)
Control sessions, 180° abduction	119	111	119	46	213	149
Control sessions, 90° abduction	119	111	119	46	215	149
Control sessions, hand-to-mouth	122	111	116	45	216	149
Gaps of at least 22 days (longest gap, days)	0 (12)	0 (1)	1 (25)	1 (23)	1 (22)	0 (16)
Use before the window: active days/sessions	113/437	32/84	69/208	92/257	0/0	142/676
Scheduled sessions per day, median (quartiles)	5 (4–6)	4 (3–5)	6 (4–6)	2 (1–6)	6 (5–6)	5 (5–6)
Scheduled sessions completed, % (with deleted assignments counted)	47.8 (42.2)	46.3 (37.8)	12.8 (12.8)	27.4 (18.0)	48.6 (47.3)	93.1 (89.6)

Days are counted inclusively; months are the time from the first to the last day (days minus one) divided by 30.44, the time scale of the models (Section 2.7). Recorded time is the sum of the step timers of completed sessions (Section 2.6); repetitions are those counted by the application for either arm (Section 2.4). Control-session counts are after the exclusions described in Section 2.5. Scheduled sessions are the assignments scheduled for a day of the window, without those that were deleted and not completed.

**Table 4:** Leading cues in the control sessions of the observation window: mean cue rate, % (share of control sessions in which the cue was shown at least once, %), and share of control sessions without cue records, %.

	180° Abduction, Elbow Cue		90° Abduction, Elbow Cue		Hand-to-Mouth, Shoulder Cue	
Patient	Cue Rate (with Cue)	No Records	Cue Rate (with Cue)	No Records	Cue Rate (with Cue)	No Records
P1	61.3 (90.8)	0.8	18.8 (76.5)	0.8	50.5 (99.2)	1.6
P2	70.4 (100.0)	0.0	13.0 (44.1)	21.6	57.3 (90.1)	9.9
P3	48.7 (99.2)	0.8	17.7 (79.8)	2.5	25.4 (69.8)	20.7
P4	45.6 (100.0)	0.0	3.5 (45.7)	39.1	1.9 (26.7)	71.1
P5	89.6 (100.0)	0.5	54.0 (99.1)	0.0	5.3 (29.6)	67.6
P6	73.4 (100.0)	0.0	13.3 (89.9)	1.3	33.2 (89.9)	6.0

The cue rate is the share of repetitions in which the cue was counted, averaged over the control sessions. The numbers of control sessions are given in Table 3.

**Table 5:** Within-patient change in the rate of the leading cue: model-based difference between the last and the first control session, percentage points [95% CI]. For series with a stable category, the estimate and its CI are printed in bold, followed by the category.

Patient	180° Abduction, Elbow Cue	90° Abduction, Elbow Cue	Hand-to-Mouth, Shoulder Cue
P1	**74.7 [59.2; 90.2]** increase	**−32.3 [−50.6; −13.9]** decrease	15.6 [6.9; 24.3] ^a^
P2	49.9 [30.8; 69.1] ^a^	11.0 [0.3; 21.6] ^a^	21.2 [−13.7; 56.1] ^a^
P3	−13.6 [−24.3; −3.0] ^a^	−8.6 [−13.9; −3.2] ^a^	22.6 [7.7; 37.5] ^a^
P4	−28.3 [−46.3; −10.4] ^b^	1.4 [−4.0; 6.7] ^b^	too few events
P5	−12.2 [−18.4; −5.9] ^a^	−14.2 [−32.7; 4.4] ^a^	not categorised
P6	**−61.7 [−76.1; −47.3]** decrease	**2.2 [−2.3; 6.6]** no marked change	−36.7 [−61.5; −11.9] ^a^

^a^ The category changes in at least one applicable sensitivity analysis. ^b^ A sensitivity analysis could not be evaluated.

**Table 6:** Within-patient change in the time per repetition: model-based change between the last and the first control session, % [95% CI]. For series with a stable category, the estimate is printed in bold and followed by the category.

Patient	180° Abduction	90° Abduction	Hand-to-Mouth
P1	0.2 [−20.6; 26.5] ^a^	−24.1 [−34.9; −11.5] ^a^	6.5 [−4.2; 18.4] ^a^
P2	−15.1 [−26.1; −2.6] ^a^	−7.7 [−17.9; 3.8] ^a^	−29.0 [−44.3; −9.4] ^a^
P3	−19.9 [−34.0; −2.8] ^a^	2.4 [−32.6; 55.7] ^a^	**11.7 [−27.4; 71.8]** undetermined
P4	43.2 [18.8; 72.7] ^b^	48.0 [19.1; 83.9] ^b^	44.9 [11.8; 87.8] ^b^
P5	14.3 [−0.4; 31.0] ^a^	−21.3 [−28.1; −13.8] ^a^	**−39.4 [−50.7; −25.5]** faster
P6	**−9.1 [−19.7; 2.8]** undetermined	9.5 [−1.6; 21.7] ^a^	1.8 [−15.0; 21.9] ^a^

^a^ The category changes in at least one applicable sensitivity analysis. ^b^ A sensitivity analysis could not be evaluated.

**Table 7:** Technical check of the deployed 90° rules in healthy adults simulating compensations (621 repetitions, 18 participants): repetitions cued between their start and the count at 78° against the agreed label of two clinicians for the landmarks as stored (16:9) and on a 4:3 crop; estimate [95% CI].

Pattern (Pos./Eval.)	Image	TP/FP/FN/TN	Sensitivity	Specificity	PPV	NPV	F1
Elbow flexion	16:9	37/4/45/529	0.45 [0.27; 0.64]	0.99 [0.98; 1.00]	0.90 [0.74; 1.00]	0.92 [0.90; 0.95]	0.60 [0.41; 0.76]
(82/615)	4:3	28/1/54/532	0.34 [0.18; 0.52]	1.00 [0.99; 1.00]	0.97 [0.87; 1.00]	0.91 [0.89; 0.93]	0.50 [0.31; 0.68]
Shoulder elevation	16:9	0/1/107/455	0/107 ^a^	1.00 [0.99; 1.00]	0/1 ^b^	0.81 [0.76; 0.85]	0.00
(107/563)	4:3	0/7/107/449	0/107 ^a^	0.98 [0.97; 1.00]	0/7 ^b^	0.81 [0.76; 0.84]	0.00
Trunk lean	16:9	38/34/48/496	0.44 [0.26; 0.63]	0.94 [0.91; 0.96]	0.53 [0.37; 0.65]	0.91 [0.89; 0.94]	0.48 [0.31; 0.62]
(86/616)	4:3	48/51/38/479	0.56 [0.36; 0.75]	0.90 [0.88; 0.93]	0.48 [0.36; 0.58]	0.93 [0.90; 0.96]	0.52 [0.36; 0.65]
Head tilt	16:9	0/0/99/510	0/99 ^a^	1.00 [1.00; 1.00]	— ^c^	0.84 [0.82; 0.86]	0.00
(99/609)	4:3	6/1/93/509	0.06 [0.00; 0.14]	1.00 [0.99; 1.00]	6/7 ^b^	0.85 [0.82; 0.87]	0.11 [0.00; 0.24]
Asymmetry (104/537)		no deployed cue ^d^					

Pos./eval.—repetitions rated as showing the pattern by both clinicians/repetitions on which they agreed for that pattern; 16:9—landmarks as stored; 4:3—centred crop of the same height, emulating the default camera image of the application; TP, FP, FN, TN—true positives, false positives, false negatives, true negatives; PPV, NPV—positive and negative predictive value; F1—harmonic mean of sensitivity and PPV (0.00 without interval where no positive repetition was cued). CIs from 2000 bootstrap resamples of participants. ^a^ No positive repetition cued; upper 95% limit 0.035 (shoulder) and 0.038 (head) without, and about 0.10 with, allowance for clustering. ^b^ Fewer than 20 cued repetitions: given as a count; Wilson 95% CI, without allowance for clustering, [0.00; 0.79] (0/1), [0.00; 0.35] (0/7) and [0.49; 0.97] (6/7). ^c^ Never cued. ^d^ The deployed rules have no cue for inter-limb asymmetry.

## Data Availability

No videos of the training sessions were recorded. The data supporting the findings contain personal data and are not publicly available; aggregated data and the analysis script are available from the corresponding author upon reasonable request. The source code of the application is not publicly available; the cue rules are described in Section 2.4. The frame-level landmarks from our previous study used in the technical check are not publicly available (the public release [53] contains repetition-level data); they and the replay script are available from the corresponding author upon reasonable request.

## Abbreviations

The following abbreviations are used in this manuscript:

ARAT	Action Research Arm Test
CI	Confidence interval
DALY	Disability-adjusted life year
HAC	Heteroskedasticity- and autocorrelation-consistent
ICF	International Classification of Functioning, Disability and Health
MCA	Middle cerebral artery
MRC	Medical Research Council
mRS	Modified Rankin Scale
NPV	Negative predictive value
pp	Percentage points
PPV	Positive predictive value
RECORD	REporting of studies Conducted using Observational Routinely collected health Data
RGB	Red–green–blue (video)
RGB-D	Red–green–blue–depth (camera)
SA1–SA7	Sensitivity analyses 1–7
STROBE	Strengthening the Reporting of Observational Studies in Epidemiology
TIDieR	Template for Intervention Description and Replication
TP, FP, FN, TN	True positives, false positives, false negatives, true negatives
VB	Vertebrobasilar territory
WHO	World Health Organization
